# Treatment of Primary Pulmonary Aspergillosis: An Assessment of the Evidence

**DOI:** 10.3390/jof2030025

**Published:** 2016-09-08

**Authors:** Ethan R. Stewart, George R. Thompson

**Affiliations:** 1 Department of Internal Medicine, Division of Infectious Diseases, Davis Medical Center, 4150 V Street, Suite G500, Sacramento, CA 95817, USA; ethanstewart@gmail.com; 2 Department of Medical Microbiology and Immunology, University of California, Rm. 3138, Tupper Hall, One Shields Ave, Davis, CA 95616, USA

**Keywords:** aspergillosis, treatment, voriconazole, isavuconazole, combination therapy, posacaonzole, echinocandins

## Abstract

*Aspergillus* spp. are a group of filamentous molds that were first described due to a perceived similarity to an aspergillum, or liturgical device used to sprinkle holy water, when viewed under a microscope. Although commonly inhaled due to their ubiquitous nature within the environment, an invasive fungal infection (IFI) is a rare outcome that is often reserved for those patients who are immunocompromised. Given the potential for significant morbidity and mortality within this patient population from IFI due to *Aspergillus* spp., along with the rise in the use of therapies that confer immunosuppression, there is an increasing need for appropriate initial clinical suspicion leading to accurate diagnosis and effective treatment. Voriconazole remains the first line agent for therapy; however, the use of polyenes, novel triazole agents, or voriconazole in combination with an echinocandin may also be utilized. Consideration as to which particular agent and for what duration should be made in the individual context for each patient based upon underlying immunosuppression, comorbidities, and overall tolerance of therapy.

## 1. Introduction

Invasive fungal infections (IFIs) are responsible for significant morbidity and mortality, particularly in immunocompromised hosts. In this population, molds and specifically invasive aspergillosis, have been found responsible for the increasing number of deaths from IFIs [1,2,3,4]. The number of patients “at risk” for mold infections continues to increase with more intensive and prolonged chemotherapeutic regimens, immunosuppressive practices, and novel immunotherapeutic agents.

Significant advances in our understanding of the epidemiology, clinical, and radiographic manifestations of invasive aspergillosis have occurred, enabling further refinement of diagnostic testing in different patient populations. The highest incidence of infection occurs in allogeneic hematopoietic stem cell transplant recipients, although others including solid organ transplant recipients and those with qualitative or quantitative granulocyte deficits, within the intensive care unit, or with structural lung disease (e.g., preexisting cavities) are also at risk [2,5,6]. The majority of infections are caused by *Aspergillus fumigatus*, *A. flavus*, *A. niger*, or *A. terreus*. Improvements in treatment have also occurred with the availability of new antifungal agents and formulations, exhibiting improvements in pharmacokinetic parameters and toxicity [7]. A recent study evaluating combination therapy has also been completed, further expanding treatment options for patients with invasive pulmonary aspergillosis (IPA) [8]. A number of novel agents are in various stages of clinical development (F2G and novel glucan synthase inhibitors, among others) and may offer additional advantages over currently available agents.

This review will discuss the currently recommended agents for the treatment of invasive aspergillosis, examine the results from key clinical trials, and outline the concerns that arise during the care and follow-up of patients with this morbid infection.

## 2. Primary Treatment

The Infectious Diseases Society of America (IDSA) guidelines for the diagnosis and management of aspergillosis have been recently updated (Table 1) [9]. Patients with a disease presentation consistent with invasive aspergillosis should have antifungal treatment initiated while a formal diagnostic workup is completed.

### 2.1. First-Line Therapy

#### Voriconazole

Voriconazole remains the preferred agent for the treatment of aspergillosis based on the pivotal study of Herbrecht et al. [10]. This study was an unblinded randomized trial, designed to evaluate the efficacy and safety of voriconazole (two doses of 6 mg/kg on Day 1, then 4 mg/kg twice daily for at least seven days, followed by 200 mg orally twice daily or intravenous compared to amphotericin B deoxycholate (1–1.5 mg/kg per day) as primary therapy for invasive aspergillosis. All patients in this study exhibited proven or probable diseases. In this study, a complete or partial response was considered a successful outcome.

One hundred forty-four patients received voriconazole, while 133 received amphotericin B deoxycholate. The majority of patients in this study were hematopoietic stem-cell transplant recipients, had acute leukemia, or had another hematologic disorder. Twelve weeks after the initiation of antifungal therapy, successful responses were observed in 52.8% of voriconazole-treated patients (complete responses, 20.8%; partial responses, 31.9%) and 31.6% of amphotericin-B-treated patients (complete responses, 16.5%; partial responses 15.0%). This absolute difference (52.7% − 31.5% = 21.2%) had a confidence interval (CI) of 10.4–32.9.

Most importantly, the survival rate at 12 weeks was 70.8% in the voriconazole group and 57.9% in the amphotericin B group (HR, 0.59; 95% CI, 0.40–0.88). Patients receiving voriconazole had fewer overall drug-related adverse events, although visual disturbance was common in the voriconazole-treated group (44.8%).

Subsequent publications have found these visual disturbances to range from changes in light perception and seeing “flashing lights” to visual hallucinations [11,12]. These effects are transient, reversible with discontinuation of therapy, generally occur 30 min after the receipt of therapy, last for ~30 min, and occur in roughly 30% of patients [13].

### 2.2. Alternative First-Line Agents

Alternative therapeutic options include isavuconazole, voriconazole plus an echinocandin (combination therapy), liposomal amphotericin B, and other lipid formulations of amphotericin B.

#### 2.2.1. Isavuconazole

Isavuconazole is a new mold-active triazole agent with activity against the Mucorales, *Aspergillus* spp., and yeasts [14,15]. A Phase 3 double-blind randomized study comparing isavuconazole (200 mg IV (intravenous) three times daily for Days 1 and 2, followed by 200 mg once daily IV (intravenous) or orally) to voriconazole (6 mg/kg IV twice daily on Day 1, followed by 4 mg/kg IV twice daily on Day 2, then either 4 mg/kg IV twice daily or 200 mg oral twice daily from Day 3 onwards) in the treatment of invasive aspergillosis has recently been completed [16]. The primary endpoint was all-cause mortality at Day 42 in the intention-to-treat (ITT) population. The main secondary endpoint was overall response to therapy. Similar to the aforementioned Herbrecht study [10], the majority of patients in this study had underlying hematologic malignancy.

All-cause mortality through Day 42 for the ITT population was 18.6% in the isavuconazole-treated group and 20.2% in the voriconazole-treated group (treatment difference: −1.6%; 95% CI: −7.8 to 5.7) confirming the non-inferiority of isavuconazole. The overall response rates at the end of treatment (EOT) were 35.0% in the isavuconazole group and 36.4% in the voriconazole group. Additionally, isavuconazole had significantly fewer study drug-related adverse events than did voriconazole (42.4% vs. 59.8%; *p* < 0.001). This 17% difference in drug-related adverse events (AEs) were primarily the lower rates of hepatobiliary disturbance, photosensitivity, and visual changes in the isavuconazole-treated group, although it is still recommended to monitor liver function tests for patients receiving isavuconazole due to the possibility of hepatobiliary disturbance while on antifungal therapy.

#### 2.2.2. Combination Therapy

Combination therapy has been supported by generally favorable in vitro and in vivo data [17,18,19]. These studies have examined polyenes and mold-active triazoles with echinocandins. Clinical data has followed and a recently completed randomized trial compared voriconazole monotherapy to combination therapy with voriconazole plus anidulafungin [8]. This trial enrolled 454 patients with a hematologic malignancy in an attempt to assess the possible superiority of combination therapy on 6-week survival. Mortality after 6 weeks was 19.3% for those receiving combination therapy, and 27.5% for monotherapy recipients (*p* = 0.087, 95% CI: −19 to 1.5). Secondary mortality benefits favored combination therapy. In a post-hoc analysis, patients diagnosed with probable aspergillosis (the majority of patients in the study), had the largest effect on mortality (15.7% combination vs. 27.3% monotherapy, *p* = 0.037, 95% CI, −22.7 to −0.4). This study was underpowered to assess treatment superiority and it seems likely that combination therapy has a role in the treatment of invasive aspergillosis. For this reason, the newly updated IDSA aspergillosis guidelines recommend considering combination therapy in the setting of severe disease, particularly in patients with hematologic malignancy.

Other drug combinations are of interest and may be needed in selected clinical settings, although they have not been conducted in the rigorous fashion of more recent Phase 3/4 trials. In a small study, 30 patients with underlying hematologic malignancy and proven or probable invasive aspergillosis were randomized to liposomal amphotericin B (L-AMB) (3 mg/kg/day) plus caspofungin or high dose L-AMB alone (10 mg/kg/day). There were more complete or partial responses in the combination group at the end of therapy (67% vs. 27%, *p* < 0.03) and a trend towards a greater response at 12 weeks (80% vs. 67%). Survival at 12 weeks was 100% vs. 80%, respectively, but all deaths were due to progressive hematologic disease, and in only one was invasive aspergillosis deemed to be contributory. Interestingly, elevated creatinine was more frequently seen in the monotherapy group (23% vs. 7%) [20]. Given the availability of alternative agents with lower toxicity profiles, it seems unlikely that a larger study will be conducted.

Other drug combinations appear promising [21], but clinical trials have yet to be undertaken to subject these to the same level of scrutiny. Additional questions of optimal drug combinations, optimal drug dosing, pharmacokinetic interactions, potential toxic interactions or in vivo antagonism, and cost-benefit ratios of primary combination antifungal therapy require further investigation.

#### 2.2.3. Liposomal Amphotericin B

Another alternative agent as primary therapy is liposomal amphotericin B (L-AMB). L-AMB has been evaluated in multiple small randomized trials and demonstrated efficacy although the most compelling data demonstrating effectiveness was observed in the AmBiLoad Trial [22]. This study followed animal data that suggested higher doses of liposomal amphotericin B could improve outcomes [23].

The AmBiLoad trial was a double-blind study evaluating the response to L-AMB at either 3 or 10 mg/kg per day for 14 days followed by 3 mg/kg per day. The primary end point was a favorable (complete or partial) response at the end of the study treatment. Although this study included all patients with invasive mold infections, aspergillosis accounted for 97% of the total cases. A favorable response was seen in 50% vs. 46% of patients in the 3- and 10-mg/kg per day groups, respectively (treatment difference 4%; 95% CI, −10% to 18%; *p* > 0.05), and survival rates at 12 weeks were 72% and 59%, respectively. Higher rates of nephrotoxicity and hypokalemia were seen in the 10 mg/kg per day group. Therefore, there appears to be no benefit, and significant harm, with the higher L-AMB dosing strategy in the treatment of invasive aspergillosis.

#### 2.2.4. Other Lipid Amphotericin B Formulations

Other lipid formulations of amphotericin B have been evaluated in smaller studies. A single randomized trial evaluated amphotericin B colloidal dispersion formulation (ABCD) at 6 mg/kg/day to amphotericin B deoxycholate (AmB) at 1 or 1.5 mg/kg/day for the treatment of patients with invasive aspergillosis. A total of 174 patients were enrolled and response rates were not found different between groups—52% in the ABCD group and 51% in the AmB group (*p* = 0.96)—and morality differences were also not observed (36% vs. 45%; *p* = 0.4) [24]. Infusion related reactions were more common in the ABCD group, although renal toxicity was less common. ABCD is not recommended unless other therapeutic options are unavailable.

Another lipid AmB alternative is ABLC (5 mg/kg/day), which has not been studied in randomized trials for IA, but has been reported to be effective in observational studies, particularly in the setting of salvage infection [25,26,27,28]. ABLC is generally well-tolerated compared with AmB, but, similar to ABCD, is not generally used given the availability of other agents.

#### 2.2.5. Echinocandins

Primary therapy with an echinocandin as monotherapy should not be used. This class had had limited evaluation in the treatment of aspergillosis, although caspofungin has exhibited efficacy in small non-comparative studies of drug administered for both primary and “salvage” therapy [29,30,31,32,33,34,35]. The updated IDSA guidelines do not support the use of echinocandins as monotherapy based on a lack of robustly powered comparative trials. In the rare situation where both triazoles and polyenes are contraindicated, an echinocandin (micafungin or caspofungin) can be used. Anidulafungin has not been sufficiently evaluated for inclusion.

#### 2.2.6. Posaconazole

Posaconazole is available as an oral suspension, delayed-release tablet and intravenous formulation, but has been studied primarily as primary prophylaxis against invasive aspergillosis [36,37]. Primary therapy of IA with posaconazole is not recommended due to the lack of proven efficacy as primary therapy. An industry sponsored study designed to evaluate the safety and efficacy of posaconazole versus voriconazole for the treatment of invasive aspergillosis is currently ongoing (ClinialTrials.gov NCT01782131). At this time, posaconazole remains a useful agent in the salvage setting [38].

#### 2.2.7. Itraconazole

Itraconazole is available as an oral solution or capsule, while intravenous intraconazole has limited availability. Itraconazole is used primarily as a corticosteroid sparing agent in non-invasive forms of aspergillosis or as an alternative agent to posaconazole in the prophylaxis against IA [9]. In a prior study evaluating itraconazole in the treatment of invasive aspergillosis, only 39% had a complete or partial response [39]. Given the availability of other agents, the poor bioavailability of itraconazole and multiple drug–drug interactions, itraconazole should be used in the treatment of invasive aspergillosis only if all other options have been exhausted.

## 3. Duration of Therapy

The duration of therapy for invasive aspergillosis has not been well-defined; however, it is generally recommended that treatment be continued for a minimum of 6–12 weeks [9]. The duration and degree of immunosuppression, the site and burden of disease, and demonstration of symptomatic improvement should all be considered prior to the cessation of therapy. The decision to stop therapy should follow serial clinical examination of all signs and symptoms, and follow-up radiographic imaging.

The natural history of invasive aspergillosis in the immunosuppressed patient can complicate follow-up, and it is important to recall the volume of pulmonary infiltrates may increase for the first 7–10 days of therapy, especially in the context of granulocyte recovery [40]. The use of serial serum GM (galactomannan) assays for therapeutic monitoring is promising but remains investigational. However, increases in *Aspergillus* antigen levels signify a poor prognosis.

Patients exhibiting a response to therapy should receive secondary prophylaxis if subsequent immunosuppression is anticipated. Secondary prophylaxis is maintained for the duration of immunosuppression in an attempt to prevent disease recurrence.

## 4. Adjunctive Therapy

### 4.1. Medical Therapy

Attempts to ameliorate underlying immunologic derangements that predispose to invasive fungal infections are of paramount importance. Withholding chemotherapy until clinical improvement, reducing or stopping immunosuppressive medications, and the correction of underlying comorbidities that predispose to invasive disease (e.g., diabetic ketoacidosis) are all essential. In the neutropenic patient, the use of colony stimulating factors (granulocyte-colony stimulating factor (G-CSF) or granulocyte macrophage-colony stimulating factor (GM-CSF)) are recommended in attempts to minimize the duration of neutropenia.

Granulocyte transfusions are frequently used as adjunctive therapy; however, there are potential harms to this approach with acute lung injury observed in past studies [41]. A recently completed study evaluated neutropenic patients who received standard antimicrobial therapy vs. standard antimicrobial therapy plus daily granulocyte transfusions and dexamethasone [42]. The overall success rate at Day 42 was no different between groups, with 42% vs. 43% (*p* > 0.99) of patients exhibiting a successful response to treatment.

### 4.2. Surgery

Surgical intervention is indicated in patients with invasive aspergillosis contiguous with critical structures such as the great vessels (aorta, vena cava) or critical organs. Recalcitrant or massive hemoptysis from a single focus, or lesions within bones, also frequently requires surgical intervention. From retrospective studies, it appears surgical intervention is also important in the control of invasive fungal sinusitis [43], endocarditis [44,45], or focal central nervous system disease [46]. Localized cutaneous disease also frequently benefits from surgical debridement.

## 5. Conclusions and Future Directions

Invasive aspergillosis is an extremely morbid infection of increasing significance given the wide-spread use of many immunosuppressive agents for a variety of clinical purposes. While voriconazole monotherapy remains the primary therapeutic option, alternatives such as liposomal amphotericin B, isavuconazole, and voriconazole with an echinocandin as combination therapy are reasonable primary alternatives in the appropriate clinical setting. Echinocandins alone as monotherapy are generally avoided except in salvage therapy or when there are contraindications of primary alternatives. Duration of therapy, which should be individualized based upon degree of immunosuppression and site of infection, however, is often for at least 6–12 weeks. Surgical intervention should be adjunctively considered for recalcitrant disease or invasion of vascular structures, critical organs, bone, or local cutaneous disease. Important directions for future research should evaluate an appropriate duration of therapy in addition to examining the efficacy of newer pharmaceutical agents. Additional optimization of current drugs in combination for the purpose of maximizing therapeutic effect while minimizing toxicities would be another important avenue of study.

## Figures and Tables

**Table 1:** Treatment recommendations for invasive aspergillosis.

Recommendation	Drug	Dosing	Comments
Primary	Voriconazole	6 mg/kg IV every 12 h times two then 4 mg/kg IV every 12 h	Oral therapy at mg/kg dosing or 200–300 mg every 12 h; TDM required
Alternatives	Lipsosomal amphotericin B (L-AMB)	3–5 mg/kg/day IV	
	Isavuconazole	200 mg every 8 h IV or PO times six then 200 mg daily IV or PO	Need for TDM remains undefined
	Voriconazole plus Anidulafungin	Vorizonazole as above plus Anidulafungin 200 mg IV daily times one then 100 mg IV daily	Combination therapy considered in severe disease and with hematologic malignancy
	Amphotericin B Lipid Complex (ABLC)	5 mg/kg/day IV	
Secondary	Caspofungin	70 mg IV daily times one then 50 mg IV daily	Monotherapy as salvage
	Posaconazole	Oral suspension: 200 mg PO every 8 h, Tablet: 300 mg PO every 12 h times two then 300 mg PO daily, Intravenous: 300 mg IV every 12 h times two then 300 mg IV daily	Caution in use of tablet formulation with acid suppression; TDM required
	Itraconazole	200 mg PO every 12 h	TDM required
