# Correction: Stewart, E.R.; Thompson, G.R. Treatment of Primary Pulmonary Aspergillosis: An Assessment of the Evidence. *J. Fungi* 2016, *2*, 25.

**DOI:** 10.3390/jof2040027

**Published:** 2016-11-04

**Authors:** Ethan R. Stewart, George R. Thompson

**Affiliations:** 1 Department of Internal Medicine, Division of Infectious Diseases, Davis Medical Center, 4150 V Street, Suite G500, Sacramento, CA 95817, USA; ethanstewart@gmail.com; 2 Department of Medical Microbiology and Immunology, University of California, Rm. 3138, Tupper Hall, One Shields Ave, Davis, CA 95616, USA

The authors of the published paper [1] would like to correct Table 1. The sixth row in the second column should have been Amphotericin B Lipid Complex (ABLC). Therefore, Table 1 should read as follows:

We apologize for any inconvenience caused to readers. The manuscript will be updated and the original will remain available on the article webpage.

## Figures and Tables

**Table 1:** Treatment recommendations for invasive aspergillosis.

Recommendation	Drug	Dosing	Comments
Primary	Voriconazole	6 mg/kg IV every 12 h times two then 4 mg/kg IV every 12 h	Oral therapy at mg/kg dosing or 200–300 mg every 12 h; TDM required
Alternatives	Lipsosomal amphotericin B (L-AMB)	3–5 mg/kg/day IV	
	Isavuconazole	200 mg every 8 h IV or PO times six then 200 mg daily IV or PO	Need for TDM remains undefined
	Voriconazole plus Anidulafungin	Vorizonazole as above plus Anidulafungin 200 mg IV daily times one then 100 mg IV daily	Combination therapy considered in severe disease and with hematologic malignancy
	Amphotericin B Lipid Complex (ABLC)	5 mg/kg/day IV	
Secondary	Caspofungin	70 mg IV daily times one then 50 mg IV daily	Monotherapy as salvage
	Posaconazole	Oral suspension: 200 mg PO every 8 h, Tablet: 300 mg PO every 12 h times two then 300 mg PO daily, Intravenous: 300 mg IV every 12 h times two then 300 mg IV daily	Caution in use of tablet formulation with acid suppression; TDM required
	Itraconazole	200 mg PO every 12 h	TDM required
